# Superolateral Hoffa fat pad edema in adolescent competitive alpine skiers: temporal evolution over 4 years and risk factors

**DOI:** 10.1186/s13244-024-01633-8

**Published:** 2024-02-16

**Authors:** Georg C. Feuerriegel, Adrian A. Marth, Stefan Fröhlich, Johannes Scherr, Jörg Spörri, Reto Sutter

**Affiliations:** 1 Department of Radiology, Faculty of Medicine, Balgrist University Hospital, University of Zurich, Zurich, Switzerland; 2 Swiss Center for Musculoskeletal Imaging, Balgrist Campus AG, Zurich, Switzerland; 3 Sports Medical Research Group, Department of Orthopedics, Balgrist University Hospital, University of Zurich, Zurich, Switzerland; 4 University Centre for Prevention and Sports Medicine, Department of Orthopedics, Balgrist University Hospital, University of Zurich, Zurich, Switzerland

**Keywords:** Magnetic resonance imaging, Edema, Athletes, Knee injuries

## Abstract

**Objectives:**

To longitudinally assess and correlate the prevalence of superolateral Hoffa fat pad (SHFP) edema with changes in features of the knee extensor mechanism in adolescent competitive alpine skiers over 48 months.

**Methods:**

Competitive alpine skiers were prospectively enrolled in 2018 and underwent bilateral knee MRI at baseline and after 48 months. MRI was assessed for the prevalence of SHFP edema. Features of the knee extensor mechanism were assessed by measuring the trochlear sulcus angle and depth, lateral and medial trochlear inclination, trochlear angle, patella tilt, Insall‒Salvati ratio (ISR), and patellar ligament to lateral trochlear facet (PL-T) distance. Separate logistic regression models were used to calculate the odds ratios between each measurement and the presence of SHFP edema at both time points.

**Results:**

Sixty-three athletes were included in the study (mean age 15.3 ± 1.3 years, 25 women). At baseline, 23 knees had SHFP edema, increasing to 34 knees at the 48-month follow-up. At baseline, knees with measurements in the highest quartile for ISR and lowest quartile for trochlear depth and PL-T were 9.3, 5.1, and 7.7 times more likely to show SHFP edema, respectively. At follow-up, these correlations were confirmed and additionally, knees with measurements in the highest quartile for trochlear sulcus angle and the lowest quartile for lateral trochlear inclination were 4.1 and 3.4 times more likely to show SHFP edema.

**Conclusion:**

An increased prevalence of SHFP edema in competitive alpine skiers during adolescence was associated with persistent high-riding patella, reduced patellar ligament to trochlear distance, and flattened lateral trochlear facet.

**Critical relevance statement:**

In clinical routine, assessment of the mechanical properties of the knee extensor mechanism, together with anatomical developments during adolescence, may improve the understanding and management of patellofemoral instability.

**Key points:**

• Superolateral Hoffa fat pad (SHFP) edema is a frequent cause of anterolateral knee pain but the role of predisposing factors is still debated.

• A higher prevalence of SHFP edema was associated with high-riding patella, reduced patellar ligament to trochlear distance, and flattened lateral trochlear facet.

• Understanding of the mechanical interaction and the anatomical development of the knee during adolescence provides further insight into the development of SHFP edema.

**Graphical Abstract:**

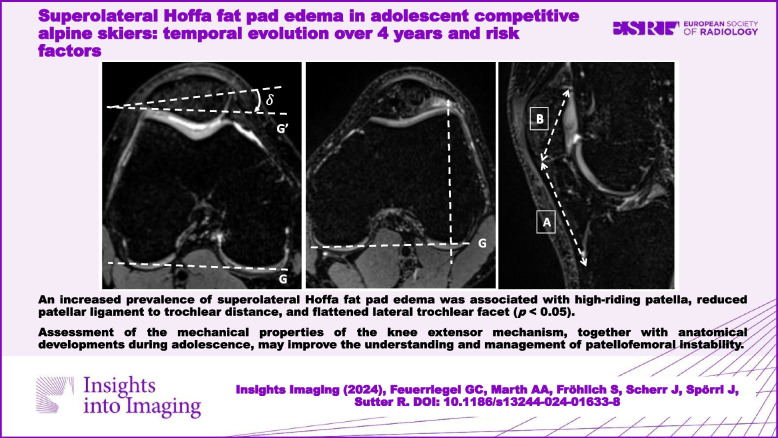

## Introduction

Superolateral Hoffa fat pad (SHFP) edema is a common finding on MRI in patients with pain in the anterior or anterolateral knee, which is commonly exacerbated by hyperextension of the knee or focal pressure on the inferior pole of the patella [1]. SHFP edema is characterized by an increased signal intensity on T2-weighted MR images in the superolateral Hoffa fat pad, which lies inferior to the patella, posterior to the patellar tendon, and anterior to the infrapatellar synovium [1, 2]. SHFP edemas are often associated with “patellar tendon-lateral femoral condyle friction syndrome,” which is caused by compression of the Hoffa fat pad between the patellar tendon and lateral femoral condyle due to overuse [2, 3]. As possible underlying causes, features of the knee extensor mechanism were identified, including the misalignment of the patellofemoral joint (laterally displaced patella and high riding patella) as well as the trochlear morphology (more anterior trochlear facet, larger trochlear sulcus angle, smaller lateral trochlear inclination angle, and increased tibial tuberosity-trochlear groove distance) [4–7]. Campagna et al. identified a short distance between the patellar ligament and the lateral trochlear facet as well as a high-riding patella to be associated with an increased prevalence of SHFP edemas [8]. Matcuk et al. successfully used the lateral patellar displacement, Insall‒Salvati ratio, and lateral trochlear inclination to create a predictive model for SHFP edema [9].

An increased prevalence of SHFP edema was found in professional athletes with higher demands for squatting and kneeling, such as competitive volleyball and beach-volleyball players [4, 10, 11]. Likewise, in competitive alpine skiing, loads of up to 1.75 body weight are exerted on one leg with the knees strongly flexed and pushed forward, resulting in extensive muscular activation of the knee extensors [12, 13]. Overuse-related knee pain is also especially frequent in young athletes, and it was shown that highly specialized young athletes are more than twice as likely to have some form of overuse complaint [14]. Growth spurts have been identified as another predisposing factor for overuse-related knee pain in young athletes, most likely due to the changed proportions and leverage caused by the rapidly growing bones and the thus altered joint loadings [15, 16]. However, to date, the longitudinal changes in the features of the knee extensor mechanism and in the patellofemoral joint alignment and their association to the prevalence of SHFP edemas have not been assessed in athletes during adolescence.

Therefore, the aim of this study was to longitudinally assess and correlate the prevalence of SHFP edema with sports-related changes in the features of the knee extensor mechanism in adolescent competitive alpine skiers over 48 months using MRI.

## Materials and methods

### Participant selection

**Fig. 1:**
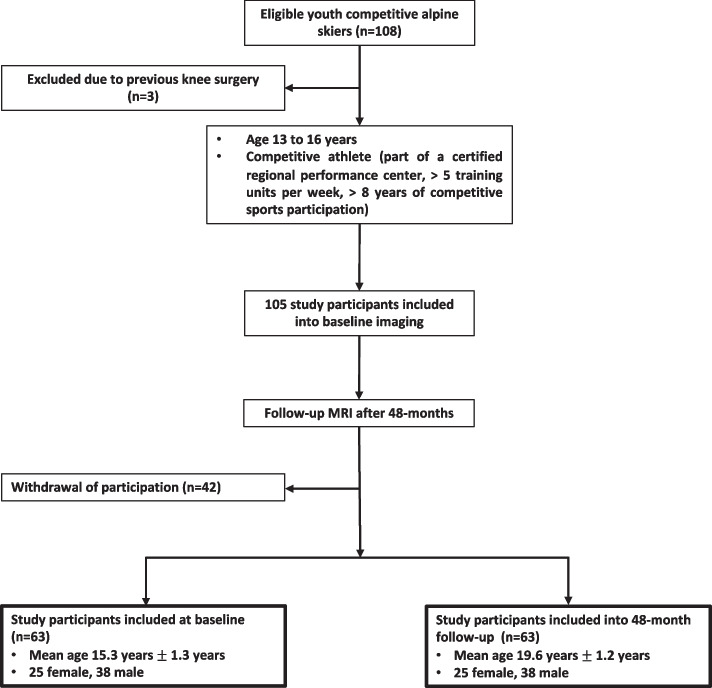
Selection flow chart for youth competitive alpine skiers

In this prospective study, adolescent competitive alpine skiers were enrolled at baseline between November 2018 and February 2019 and received bilateral 3 T MRI of the knee (*n* = 108, Fig. 1). The criteria for being characterized as a competitive alpine skier at baseline were participation in a certified regional performance center, five or more training units per week, and eight or more consecutive years of participation in competitive alpine sports. Participants with previous knee surgery or acute sports-related knee injury were excluded (*n* = 3). Between baseline and follow-up, 42 skiers withdrew their participation. Follow-up MRI of both knees was performed after 48 months in 63 participants. Participants ranged in age from 14.4 to 16.8 years at baseline and from 18.4 to 20.8 years at follow-up. Written informed consent was obtained from all study participants prior to inclusion. The underlying study protocol was approved by our institutional review board (Cantonal Ethics Committee Zurich (BASEC Nr. 2017–01395)).

### MR imaging

At baseline and 48-month follow-up, all participants were scanned with a 3 T MRI scanner (MAGNETOM Prisma; Siemens Healthcare) using a dedicated 15-channel knee coil (Tx/Rx 15-Channel Knee Coil, Siemens Healthcare). At both timepoints, a noncontrast isotropic fat-suppressed T2-weighted three-dimensional SPACE sequence was acquired for each knee. The slice position was aimed at the center of the femorotibial joint. Scan parameters: repetition time 1000 ms, echo time 108 ms, parallel imaging acceleration factor 4, acquired slice thickness 0.63 mm; field of view 160 × 160; matrix 256 × 256; receiver bandwidth, 415 Hz per pixel, leading to an acquisition time of 4 min 42 s [17].

### Image analysis

**Fig. 2:**
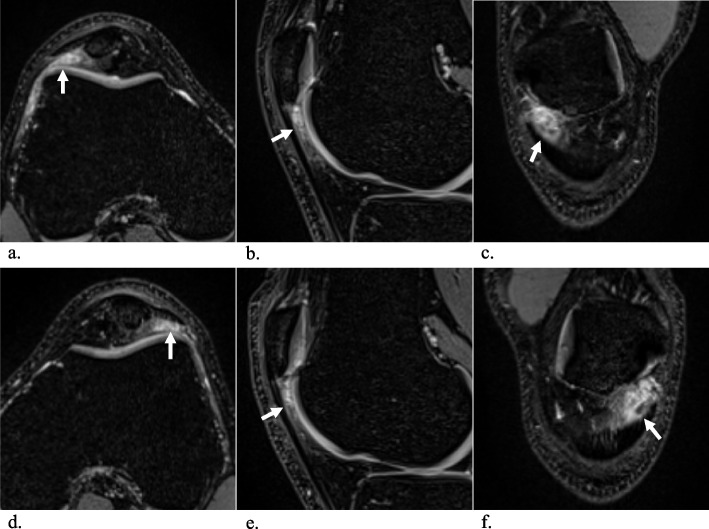
Multiplanar MRI of bilateral superolateral Hoffa fat pad (SHFP) edemas (arrows) of an 18-year-old youth competitive alpine skier (**a**–**c** axial, sagittal, and coronal images of the right knee; **d**–**f** axial, sagittal, and coronal images of the left knee). SHFP edema was defined as focal high signal intensity in the superolateral corner of the Hoffa fat pad on the fat-suppressed T2-weighted images

**Fig. 3:**
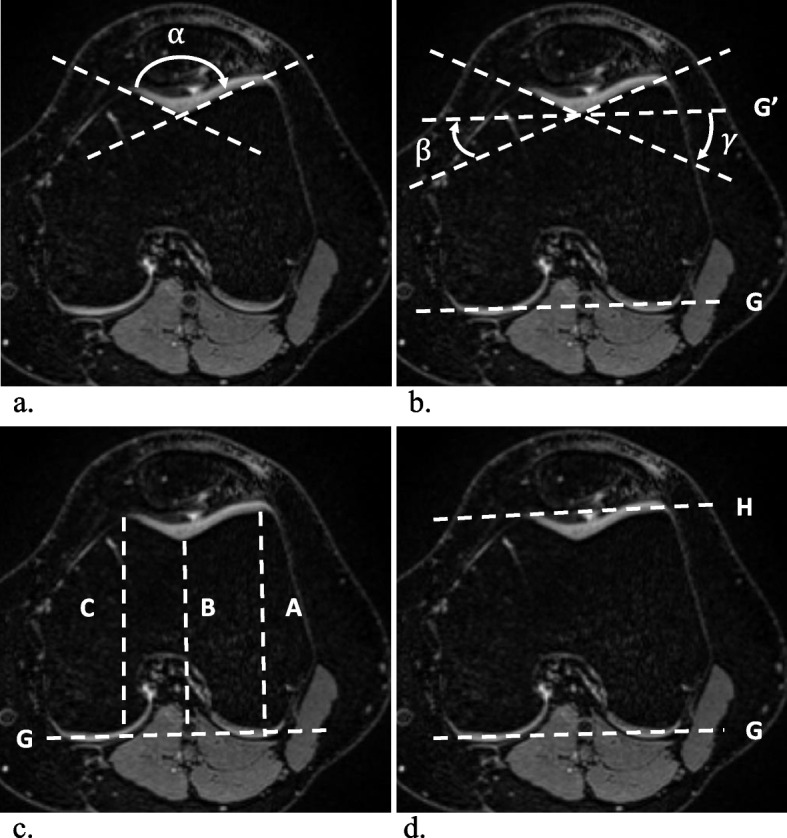
Measurements of the trochlear morphology shown on axial MR images of the left knee: The trochlear sulcus angle $\alpha$ (**a**) was defined as the angle between the medial and lateral trochlear facets. The lateral ($\beta ) and$ medial ($\gamma )$ trochlear inclination (**b**) were defined as the angles between the lateral and medial trochlear facets and the reference line (G), respectively. The trochlear sulcus depth (**c**) was measured by drawing lines perpendicular to the reference line (G) indicating the largest anterior-to-posterior diameter of the lateral (A) and medial (C) trochlear facets as well as the deepest point of the sulcus (B). Finally, trochlear depth was calculated as follows: trochlear depth in mm = *([A* + *C]/2) − B*. The trochlear angle (**d**) was defined as the angle between the reference line (G) and a line (H) drawn along the most anterior points of the medial and lateral trochlear facets

**Fig. 4:**
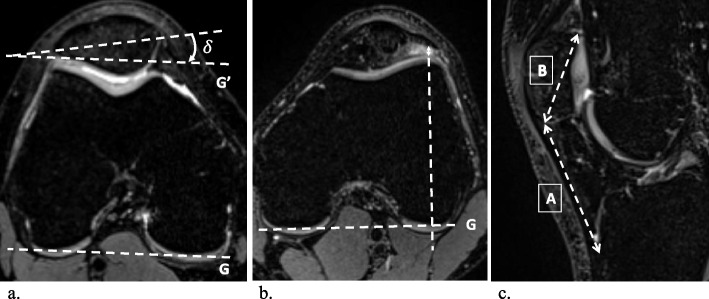
The patella tilt angle $\delta$ (**a**) was measured between the reference line (G) at the posterior femoral condyles and a line going through the medial and lateral edges of the patella at the level of the maximal width. The PL-T distance (**b**) was measured by drawing a line perpendicular to the reference line (G) which projects anteriorly through the most anterior point of the lateral trochlear facet. The distance between the dorsal aspect of the ligament and the ventral aspect of the lateral trochlear facet cartilage was measured. The patellar height is assessed with the ISR (**c**) which is defined by the ratio of the patellar tendon length (A) to the length of the patella (B). ISR, Insall‒Salvati ratio; PL-T, patella ligament to trochlear distance

**Fig. 5:**
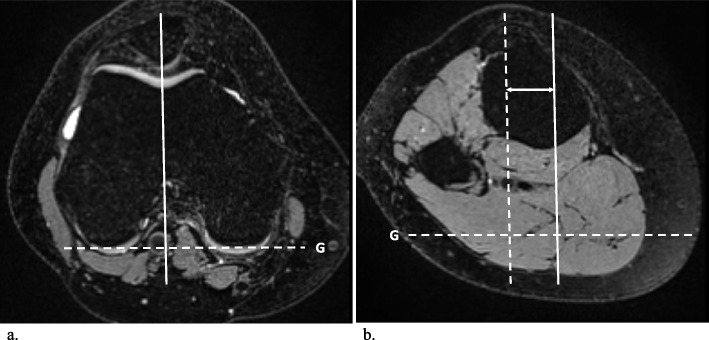
Measurements of the trochlear morphology shown on axial MR images of the right knee: the tibial tuberosity to trochlear groove distance is measured by calculating the transverse length between the trochlear groove (**a**) on the femur and tibial tuberosity (**b**) on axial images

All images were assessed by two radiologists (A.M. and G.F.) in random order, independently, separately, and blinded to all clinical information. The frequency and location of SHFP edemas were recorded for each knee, and SHFP edema was defined as focal high signal intensity on the fat-suppressed T2-weighted images located in the superolateral corner of the Hoffa fat pad (Fig. 2). Assessment of the knee extensor mechanisms was performed on axial images. The trochlear sulcus angle was measured as the angle between the medial and lateral trochlear facets. For further measurements, a reference line (G) was drawn parallel to the posterior aspect of the femoral condyles (Fig. 3) [18]. Medial and lateral trochlear inclinations were measured as the angles from the medial and lateral trochlear facets, respectively, to the reference line (G) [19, 20]. Trochlear sulcus depth was measured by drawing lines perpendicular to the reference line (G) indicating the largest anterior to posterior diameter of the lateral (A) and medial (C) trochlear facets as well as the deepest point of the sulcus (B). Finally, trochlear depth was calculated as follows: trochlear depth in mm = *([A* + *C]/2) − B* [8]. The trochlear angle was defined as the angle between the reference line (G) and a line drawn along the most anterior points of the medial and lateral trochlear facets [21]. The patellar tilt angle was measured between the reference line (G) and a line joining the medial and lateral margins of the patella (Fig. 4) [22]. To measure the shortest patellar ligament to the lateral trochlear facet (PL-T) distance, a line was drawn perpendicular to the reference line (G), which projected anteriorly through the most anterior point of the lateral trochlear facet. Afterwards, the distance between the dorsal aspect of the ligament and the ventral aspect of the lateral trochlear facet cartilage was measured on this line [8]. The Insall‒Salvati ratio (ISR) was measured on sagittal reconstructions [23, 24], and the tibial tubercle to trochlear groove (TT-TG) distance was measured as previously reported (Fig. 5) [8, 25–27]. Image analysis was performed on a picture archiving and communication system (PACS) workstation certified for clinical use (MERLIN 7.1.22, Phönix-PACS GmbH).

### Statistics

Comparison of measurements between participants with and without SHFP edema was performed using the Mann‒Whitney *U* test, and comparison between the baseline and 48-month follow-up was performed with the Wilcoxon signed-rank test. Separate logistic regression models were used to assess the relationship between each measurement of trochlear morphology and patellofemoral alignment and the presence or absence of SHFP edema at each time point. The variables were divided into quartiles and adjusted for age and sex. To assess the linear relationship of each variable to SHFP edema, they were entered as continuous variables in separate models [5]. For interreader agreement, the interclass correlation coefficient (ICC) and Cohen’s weighted $\kappa$ were used. Statistics were performed in SPSS (v. 28.0 IBM Corp.) by G. F. under supervision of an experienced biostatistician.

## Results

**Fig. 6:**
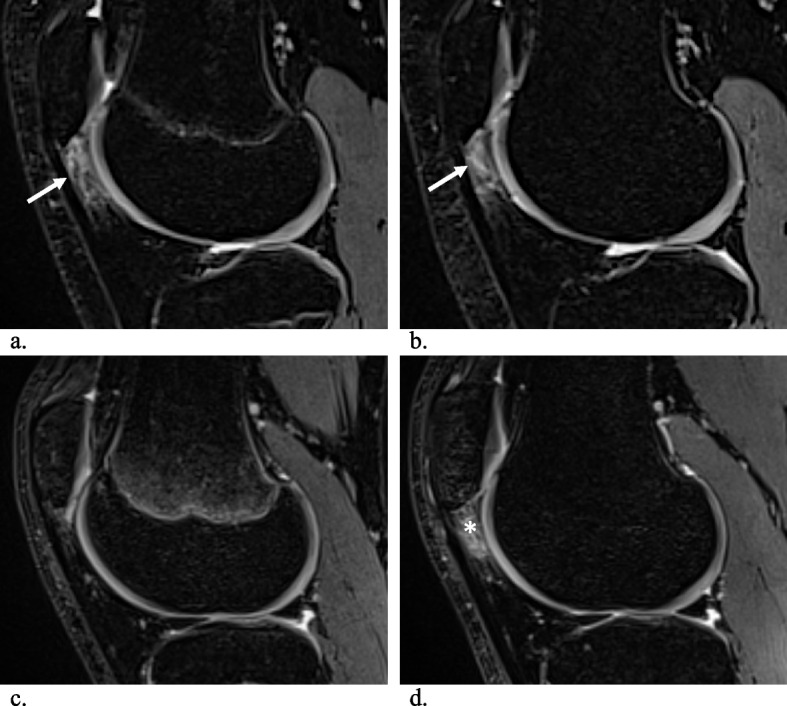
Sagittal MR images of a competitive youth alpine skier (**a** and **b**) showing persistent superolateral Hoffa fat pad edema (SHFP, arrows) of the right knee over a period of 48 months. A new SHFP edema of the left knee at the 48-month follow-up compared to baseline (asterisk) is shown at **c** and **d**

In total, 63 adolescent competitive alpine skiers were included in the study (mean age at baseline 15.6 ± 1.2 years and at follow-up 19.6 ± 1.2 years, 25 women, Table 1). At baseline, 23 knees with SHFP edema were detected (10 male and 6 female), increasing to 34 knees with SHFP edema at the 48-month follow-up (10 male, 8 female) (Fig. 6). A significant increase in bilateral affection was observed at the 48-month follow-up (baseline: 44% vs 89% follow-up, *p* = 0.03).

**Table 1:** Characteristics of the study participants

	**Baseline**	**48-month follow-up**
**Characteristics**		
Age (years)	15.2 years ± 0.6 years	19.6 years ± 1.2 years
Females^a^	25 (40%)	25 (40%)
Males^a^	38 (60%)	38 (60%)
**SHFP edema**		
Participants with SHFP edema^a^	16 (25%)	18 (29%)
Number of knees^b^	23 (37%)	34 (54%)
Bilateral^a^	7 (44%)	16 (89%)
Unilateral^a^	9 (56%)	2 (11%)
Females with SHFP edema^a^	6 (38%)	8 (44%)
Males with SHFP edema^a^	10 (62%)	10 (56%)

*SHFP* superolateral Hoffa fat pad

^a^Number and percentage of participants

^b^Number of knees and percentage

### Correlation of trochlear morphology and patellofemoral joint alignment with SHFP edema

At baseline, knees within the lowest quartile for trochlear depth and PL-T distance were 5.1 (95% CI: 1.2–9.2) and 7.7 (95% CI: 3.2–9.8) times more likely to show SHFP edema compared to knees within the highest quartile (*p* = 0.002 and *p* < 0.001 for linear trend, respectively). Compared to knees with the lowest quartile, knees within the highest quartile for ISR were 9.3 times (95% confidence interval (CI): 4.0–11.2) more likely to have SHFP edema (*p* < 0.001 for linear trend). No statistically significant associations of the trochlear sulcus angle, trochlear angle, lateral and medial trochlear inclination, TT-TG distance, and patella tilt with SHFP edema were found at baseline (Table 2).

**Table 2:** Association of measurements for trochlear morphology and patellofemoral joint alignment in quartiles with SHFP edema at baseline

**Measurement**	Quartile 1	Quartile 2	Quartile 3	Quartile 4	*p* value^1^
**Trochlear anatomy**					
**Trochlear depth**					
Range (mm)	3.01–5.37	5.38–6.01	6.02–7.00	7.01–12.21	
Odds ratio^a^	5.06 (1.21–9.27)	1.27 (0.27–5.97)	0.68 (0.12–3.73)	1.00 (REF)	0.002
**Trochlear sulcus angle**					
Range (degrees)	120.21–130.52	130.53–134.65	134.66–139.21	139.22–159.21	
Odds ratio^a^	(REF)	1.53 (0.37–6.49)	1.84 (0.46–5.35)	2.37 (0.87–7.02)	0.110
**Trochlear angle**					
Range (degrees)	0.07–1.99	2.00–3.55	3.56–5.65	5.65–8.69	
Odds ratio^a^	1.00 (0.28–3.61)	1.17 (0.34–4.06)	0.58 (0.15–2.35)	1.00 (REF)	0.886
**Lateral trochlear inclination**					
Range (degrees)	10.02–19.98	19.99–22.03	22.04–25.00	25.01–38.21	
Odds ratio^a^	3.57 (1.71–6.78)	3.12 (0.89–6.10)	2.40 (0.43–13.20)	1.00 (REF)	0.061
**Medial trochlear inclination**					
Range (degrees)	9.19–20.32	20.33–24.01	24.02–27.53	27.54–33.67	
Odds ratio^a^	1.00 (REF)	0.64 (0.01–0.54)	0.35 (0.10–1.16)	0.65 (0.20–2.14)	0.241
**TT-TG**					
Range (mm)	0.80–4.21	4.22–6.11	6.12–8.11	8.12–13.00	
Odds ratio^a^	1.00 (REF)	0.69 (0.14–3.41)	1.57 (0.39–5.32)	2.34 (0.91–7.64)	0.423
**Patella Anatomy**					
**Patella tilt**					
Range (degrees)	0.11–2.33	2.34–5.31	5.32–8.16	8.17–22.21	
Odds ratio^a^	1.00 (REF)	2.01 (0.51–7.89)	2.19 (0.57–8.35)	1.00 (0.22–4.49)	0.368
**Insall‒Salvati ratio**					
Range	0.79–1.04	1.05–1.14	1.15–1.26	1.27–1.53	
Odds ratio^a^	1.00 (REF)	3.24 (0.32–7.22)	6.14 (0.67–9.48)	9.34 (4.02–11.23)	< 0.001
**PL-T**					
Range (mm)	2.00–5.43	5.44–6.52	6.53–8.15	8.16–11.00	
Odds ratio^a^	7.69 (3.22–9.81)	5.34 (0.69–7.68)	2.45 (1.12–4.32)	1.00 (REF)	< 0.001

*REF* reference value, *SHFP* superolateral Hoffa fat pad, *TT-TG* tibial tuberosity to trochlear groove distance, *PL-T* patella ligament to trochlear distance

^1^ *p* values were calculated for the linear trend

^a^Data in brackets are given 95% confidence intervals

At follow-up again, higher odds were detected within the lowest quartile for trochlear depth (odds: 5.95 (95% CI: 1.51–9.45), *p* = 0.042 for linear trend) and PL-T distance (odds: 6.50 (95% CI: 1.96–9.60), *p* < 0.001 for linear trend) as well as for knees within the highest quartile for ISR (odds: 9.34 (95% CI: 4.02–11.23), *p* < 0.001 for linear trend, Table 3). Additionally, knees within the highest quartile for trochlear sulcus angle as well as the lowest quartile for lateral trochlear inclination were 4.1 (95% confidence interval (CI): 1.2–9.2) and 3.4 (95% CI: 0.9–11.7) times more likely to show SHFP edema (*p* = 0.035 and *p* = 0.018 for linear trend, respectively). No increased odds ratios were found for the other measurements at the 48-month follow-up.

**Table 3:** Association of measurements for trochlear morphology and patellofemoral joint alignment in quartiles with SHFP edema at the 48-month follow-up

**Measurement**	Quartile 1	Quartile 2	Quartile 3	Quartile 4	*p* value
**Trochlear anatomy**					
**Trochlear depth**					
Range (mm)	3.52–6.58	6.59–8.12	8.13–9.61	9.62–12.00	
Odds ratio^a^	5.95 (1.51–9.45)	1.45 (0.32–6.46)	1.23 (0.65–3.07)	1.00 (REF)	0.042
**Trochlear sulcus angle**					
Range (degrees)	93.24–115.21	115.22–120.41	120.42–125.56	125.57–133.52	
Odds ratio^a^	1.00 (REF)	1.55 (0.47–3.06)	1.64 (0.45–5.22)	4.19 (1.3–9.23)	0.035
**Trochlear angle**					
Range (degrees)	0.03–1.97	1.98–3.58	3.59–5.31	5.32–9.74	
Odds ratio^a^	1.82 (0.24–2.80)	2.67 (0.89–7.96)	1.00 (0.30–3.29)	1.00 (REF)	0.634
**Lateral trochlear inclination**					
Range (degrees)	17.41–25.60	25.61–29.44	29.45–34.91	34.92–49.00	
Odds ratio^a^	3.38 (0.97–11.77)	3.08 (0.82–11.53)	2.48 (0.65–9.37)	1.00 (REF)	0.018
**Medial trochlear inclination**					
Range (degrees)	15.02–27.91	27.92–31.03	31.04–34.87	34.88–44.31	
Odds ratio^a^	1.00 (REF)	0.56 (0.19–1.67)	0.58 (0.19–1.75)	0.73 (0.25–2.13)	0.128
**TT-TG**					
Range (mm)	1.52–7.76	7.77–8.53	8.54–10.76	10.77–15.23	
Odds ratio^a^	1.00 (REF)	1.76 (0.55–5.62)	1.81 (0.65–5.05)	1.39 (0.45–4.34)	0.578
**Patella Anatomy**					
**Patella tilt**					
Range (degrees)	1.23–5.05	5.06–10.23	10.24–15.87	15.88–28.97	
Odds ratio^a^	1.00 (REF)	0.89 (0.26–2.99)	2.60 (0.88–7.68)	2.00 (0.61–6.55)	0.140
**Insall‒Salvati ratio**					
Range (degrees)	0.67–1.10	1.11–1.24	1.25–1.36	1.37–1.56	
Odds ratio^a^	1.00 (REF)	2.20 (0.62–7.85)	2.64 (0.76–9.19)	5.43 (1.68–10.63)	0.002
**PL-T**					
Range (mm)	2.23–5.32	5.33–6.74	6.75–8.90	8.91–14.24	
Odds ratio^a^	6.50 (1.96–9.60)	2.71 (0.62–7.92)	0.43 (0.73–2.56)	1.00 (REF)	< 0.001

*REF* reference value, *SHFP* superolateral Hoffa fat pad, *TT-TG* tibial tuberosity to trochlear groove distance, *PL-T* patella ligament to trochlear distance

^1^ *p* values were calculated for the linear trend

^a^Data in brackets are given 95% confidence intervals

### Comparison of measurements between athletes with and without SHFP edema

At baseline and 48-month follow-up, a significantly larger trochlear depth was detected for athletes without SHFP edema compared to athletes with SHFP edema (baseline: *p* = 0.002, follow-up: *p* = 0.014, Table 4). In athletes without SHFP edema, a significantly larger PL-T distance was measured at baseline and follow-up (baseline: *p* < 0.001, follow-up: *p* < 0.001). At follow-up, significantly higher trochlear sulcus angles were found in athletes with SHFP edema compared than in athletes without edema (follow-up: *p* = 0.03), indicating a flatter trochlear surface. At baseline and follow-up athletes with SHFP edema showed higher values for ISR than in athletes without SHFP edema (baseline: *p* < 0.001, follow-up: *p* = 0.002). No significant differences between athletes with and without SHFP edema were found for the other measurements (Table 4).

**Table 4:** Comparison of mean measurements for trochlear morphology and patellofemoral joint alignment between athletes with and without SHFP edema

Measurement	Baseline		*p* value	48-month follow-up		*p* value
	**Athletes with SHFP edema**	**Athletes without SHFP edema**		**Athletes with SHFP edema**	**Athletes without SHFP edema**	
**Trochlear anatomy**						
Trochlear suclus depth^a^ (mm)	5.44 ± 1.22	6.43 ± 1.34	0.002	7.24 ± 1.47	8.00 ± 1.86	0.014
Trochlear sulcus angle^a^ (degree)	136.65 ± 6.53	134.06 ± 6.84	0.096	123.09 ± 10.98	118.59 ± 10.00	0.054
Lateral trochlear inclination^a^ (degree)	20.74 ± 3.66	22.66 ± 4.41	0.064	27.53 ± 6.69	30.78 ± 6.52	0.029
Medial trochlear inclination^a^ (degree)	22.39 ± 5.92	23.71 ± 4.44	0.491	29.53 ± 7.08	31.36 ± 5.44	0.240
Trochlear angle^a^ (degree)	3.76 ± 2.34	3.69 ± 2.21	0.889	3.49 ± 1.88	3.69 ± 2.22	0.540
TT-TG^a^ (mm)	6.59 ± 2.27	6.10 ± 2.73	0.429	8.74 ± 1.83	8.46 ± 2.70	0.445
**Patella anatomy**						
Patella Tilt angle^a^ (degree)	5.07 ± 4.35	5.97 ± 4.27	0.224	11.44 ± 5.94	9.58 ± 6.29	0.085
Insall‒Salvati ratio^a^	1.29 ± 0.15	1.11 ± 0.14	< 0.001	1.31 ± 0.14	1.19 ± 0.17	0.002
PL-T^a^ (mm)	3.51 ± 1.10	7.13 ± 1.34	< 0.001	5.26 ± 2.05	7.39 ± 2.43	< 0.001

*SHFP* superolateral Hoffa fat pad, *TT-TG* tibial tuberosity to trochlear groove distance, *PL-T* patella ligament to trochlear distance

^a^Data shown as the mean ± standard deviation

### Comparison of measurements between baseline and follow-up in athletes with SHFP edema

Comparing athletes with SHFP edema at baseline and 48-month follow-up, a significant increase was seen in the trochlear sulcus depth, lateral trochlear sulcus angle, medial trochlear sulcus angle, TT-TG distance, patella tilt angle and PL-T (*p* < 0.05, Table 5). A significant decrease was detected in the trochlear sulcus angle (baseline: 136.65 ± 6.53°, follow-up: 123.09 ± 10.98°, *p* < 0.001). No significant difference was found for the trochlear angle and ISR (*p* $\ge$ 0.05).

**Table 5:** Comparison of mean measurements for trochlear morphology and patellofemoral joint alignment in athletes with SHFP edema between baseline and 48-month follow-up

Measurement	Baseline	48-month follow-up	*p* value
**Trochlear anatomy**			
Trochlear sulcus depth^a^ (mm)	5.44 ± 1.22	7.24 ± 1.47	< 0.001
Trochlear sulcus angle^a^ (degree)	136.65 ± 6.53	123.09 ± 10.98	< 0.001
Lateral trochlear sulcus angle^a^ (degree)	20.74 ± 3.66	27.53 ± 6.69	0.006
Medial trochlear sulcus angle^a^ (degree)	22.39 ± 5.92	29.52 ± 7.08	< 0.001
Trochlear angle^a^ (degree)	3.76 ± 2.33	3.49 ± 1.88	0.132
TT-TG^a^ (mm)	6.59 ± 2.27	8.74 ± 1.83	0.01
**Patella anatomy**			
Patella tilt angle^a^ (degree)	5.07 ± 4.35	11.44 ± 5.94	0.001
Insall‒Salvati ratio^a^	1.29 ± 0.15	1.30 ± 0.14	0.685
PL-T^a^ (mm)	3.51 ± 1.10	5.26 ± 2.05	0.001

*REF* reference value, *SHFP* superolateral Hoffa fat pad, *TT-TG* tibial tuberosity to trochlear groove distance, *PL-T* patella ligament to trochlear distance

^a^Mean ± standard deviation

### Interrater agreement

The agreement for the detection of SHFP edemas was perfect with *κ* 1.00 (95% CI 1.00–1.00) and with the same number of edemas detected by both raters. The interrater agreement for the measurements of the knee extensor mechanisms and measurements of the patellofemoral joint alignment was substantial to almost perfect (trochlear depth: ICC 0.91 (95% CI 0.82–0.96) trochlear sulcus angle ICC 0.94 (95% CI 0.86–0.97), lateral trochlear inclination ICC 0.87 (95% CI 0.79–0.93) medial trochlear inclination ICC 0.86 (95% CI 0.76–0.95), trochlear angle ICC 0.92 (95% CI 0.85–0.98), patella tilt ICC 0.83 (95% CI 0.72–0.91), ISR ICC 0.89 (95% CI 0.81–0.93), PL-T distance ICC 0.81 (95% CI 0.71–0.89), and TT-TG distance ICC 0.85 (95% CI 0.79–0.95)).

## Discussion

In this study, we longitudinally assessed and correlated the prevalence of SHFP edema with sports-related changes in features of the knee extensor mechanism in a cohort of 63 adolescent competitive alpine skiers over 48 months. Over the last decade, the relationship between SHFP edema and abnormalities in the knee extensor mechanism was investigated by examining the malalignment of the patellofemoral joint with different underlying pathologies or at different levels of physical activity. However, there is only partial consensus about the underlying anatomical variations and angles associated with SHFP edema, and thus far, no longitudinal analysis has been performed [4, 5, 8, 9].

Our results revealed a SHFP edema prevalence of 25% at baseline, increasing to 29% at the 48-month follow-up. At baseline, athletes in the lowest quartile for trochlear depth and PL-T distance as well as the highest quartile for ISR demonstrated greater odds of developing a SHFP edema. In addition, athletes in the lowest quartile for lateral trochlear inclination angles and the highest quartile for trochlear sulcus angles had greater odds of developing SHFP edema at follow-up. This is in line with a previous study by Campagna et al., in which the authors noted an association of high ISR (*p* = 0.023), short PL-T distance (*p* < 0.001), and short TT-TG (*p* = 0.046) with SHFP in a cohort of 90 patients [8]. They suggested that the lateral displacement of the patella and narrowing between the patellar ligament and bone predispose an impingement of the SHFP due to the increased pressure on the SHFP during motion. Additionally, younger participants were more likely to present with SHFP edema, and the authors assumed this was due to increased physical activity [8]. High physical activity might also explain the high prevalence of 29% of athletes reported in our study, in which only competitive athletes with five or more training units per week were included. Widjajahakim et al. reported a significantly lower prevalence of 13.4% for SHFP edema in a cohort of 1134 patients aged 50 to 79 years with risk factors for osteoarthritis of the knee [5]. Similar to our study, participants with larger trochlear angles and high ISR were 1.5 and 8.9 times more likely to show SHFP edema. However, no associations were demonstrated for the lateral trochlear inclination and sulcus angle, which might be due to the changed level of physical activity or the changes associated with beginning osteoarthritis. Mehta et al. assessed the prevalence of SHFP edema and knee extensor mechanisms in a cohort of 16 competitive collegiate volleyball players aged 18–22 years and found a prevalence of 50% [4]. Similar to our study, the authors found significant differences in TT-TG distance in SHFP edema-positive athletes. The high prevalence was explained by the excessive kneeling and squatting performed during volleyball playing inducing overuse and pressure in the anterolateral knee, loading patterns that are also present in competitive alpine skiing [12, 13]. This assumption is further supported by a study by Jarraya et al., who found a prevalence of 52.1% for SHFP edema in a sample cohort of summer sports athletes participating in the 2016 Olympic games [11].

The high prevalence of SHFP edema in adolescent athletes might be better understood when assessing the development of trochlear morphology and patellofemoral joint alignment during adolescence. It was demonstrated that the lateral trochlear slope has a significant effect on the patella alignment during knee flexion and that the anterolateral femoral condyle has more contact with the patella than the medial facet, even during contraction of the quadriceps muscle [28, 29]. Furthermore, the lateral trochlea was found to be one of the most consistent anatomical determinants of trochlear morphology during maturation and it is able to compensate for changes in the slope of the medial trochlear facet, keeping the trochlear sulcus angle consistent [29, 30]. In this study, athletes with SHFP edema showed consistently lower values for lateral trochlear inclination and trochlear sulcus depth at baseline and at the 48-month follow-up than athletes without SHFP edema. The more flattened anterolateral trochlear facet predisposes the lateral displacement of the patella and increases the pressure on the SHFP during knee flexion, which favors impingement. Additionally, the consistently lower values might indicate that the development of SHFP edemas is not exclusively caused by overuse but also due to altered or delayed trochlear development, which affects patellofemoral alignment from adolescence. Therefore, the assessment of the mechanical relationship between the patella and lateral trochlea together with anatomical development is key to understanding patellofemoral instability.

Our study has limitations: First, only competitive alpine skiers were included in the study, which might introduce a selection bias. Second, only imaging of the knee was performed, and the relationship between the muscle volume and strength of the knee extensor muscles with SHFP edema could not be investigated. Additionally, functional aspects of the patellofemoral tracking during, e.g., kneeling could not be assessed due to the nature of the study.

In summary, an increased prevalence of SHFP edema in adolescent competitive alpine skiers was associated with a high-riding patella and a more anterolateral flattened trochlear facet. The altered anatomical proportions did not change over the course of 48 months, which highlights the importance of taking anatomical development into consideration when assessing the mechanical relationship of the patellofemoral alignment.

## Data Availability

The data that support the findings of this study are not publicly available. Data are however available from the authors upon reasonable request.

## Abbreviations

CI: Confidence interval
ICC: Interclass correlation coefficient
ISR: Insall‒Salvati ratio
MRI: Magnetic resonance imaging
PL-T: Patella ligament to trochlear distance
SHFP: Superolateral Hoffa fat pad
TT-TG: Tibial tuberosity to trochlear groove distance
